# Diagnosis of *Cytomegalovirus* infection in a very low birth weight infant using metagenomic next-generation sequencing: A case report

**DOI:** 10.1097/MD.0000000000044264

**Published:** 2025-09-05

**Authors:** Chunhui Zhao, Huimin Li, Li Guo, Menghan Jia, Jihong Liu

**Affiliations:** a Neonatology Department, Shijiazhuang Fourth Hospital, Shijiazhuang, Hebei, China; b The Unit of Pathogenic Fungal Infection & Host Immunity, CAS Key Laboratory of Molecular Virology and Immunology, Shanghai Institute of Immunity and Infection, Chinese Academy of Sciences, Shanghai, China.

**Keywords:** *Cytomegalovirus*, fever, infection, metagenomic next-generation sequencing, very low birth weight infant

## Abstract

**Rationale:**

*Cytomegalovirus* (CMV) is a DNA virus from the herpesvirus family that is widespread among humans. Very low birth weight infants (VLBWI) are particularly susceptible to postnatal CMV infection due to their compromised immune systems. The clinical manifestations of postnatal CMV infection are often nonspecific, which complicates early detection and may lead to multi-organ dysfunction and long-term sequelae.

**Patient concerns:**

A VLBWI developed unexplained persistent fever during hospitalization. Conventional diagnostic methods, including routine microbiological tests, failed to identify the causative pathogen.

**Diagnoses:**

Metagenomic next-generation sequencing (mNGS) was performed and successfully identified CMV as the etiologic agent. Traditional diagnostic approaches were insufficient, but mNGS provided a comprehensive analysis of microbial nucleic acids, leading to a definitive diagnosis.

**Interventions:**

The patient received antiviral treatment with ganciclovir following the identification of CMV by mNGS.

**Outcomes:**

After antiviral therapy, the fever resolved, and no long-term sequelae were observed during follow-up.

**Lessons:**

This case demonstrates the efficacy of mNGS as a powerful diagnostic tool for identifying the causes of unexplained infections in VLBWI. Compared with conventional methods, mNGS offers significant advantages, particularly in detecting a wide range of pathogens simultaneously. The successful diagnosis and treatment in this case underscore its clinical utility in managing complex neonatal infectious diseases.

## 1. Introduction

*Cytomegalovirus* (CMV) is a DNA herpesvirus that is widespread in the population and poses a high risk to individuals with weakened immune systems. CMV infection can be congenital, perinatal, or postnatal, with transmission routes including intrauterine infection, exposure during delivery, and postnatal transmission through breast milk, blood transfusions, or close contact.^[1]^ The transmission rates of CMV have been documented to range from 0.4% to 2.5% through congenital infections, while postnatal transmission accounts for 11.1%.^[2]^ Breast milk, rich in nutrients and immune-active components, plays a crucial role in enhancing the immunity and supporting the growth and development of very low birth weight infants (VLBWI).^[3]^ However, breast milk can also carry CMV, making it a major route for postnatal CMV (pCMV) infection. Due to the immature immune system and insufficient maternal antibody levels in extremely preterm or VLBWI, there may be a higher morbidity and mortality rate after infection with CMV.^[4]^ Reports indicate that symptomatic or severe CMV infections occur in 0% to 35% of VLBWI.^[4,5]^ Although studies report varying incidence rates of pCMV in VLBWI due to breastfeeding, they consistently highlight several risk factors: high viral load in breast milk, untreated CMV-positive breast milk, low birth weight, early infection, younger gestational age, and high levels of CMV Immunoglobulin G (IgG).^[6]^

Most VLBWI with CMV infection are asymptomatic or present with mild symptoms. However, a small proportion may suffer from multi-organ damage, including hepatitis, pneumonia, necrotizing enterocolitis (NEC), and sepsis.^[7–13]^ Recent studies have also suggested an association between pCMV infection and neurodevelopmental impairment, though long-term outcomes remain an area of ongoing investigation.^[6,14,15]^ Traditional diagnostic methods for diagnosing pCMV infection include virus isolation, serological tests, histopathological examination, and quantitative polymerase chain reaction (PCR). These methods have limitations, such as virus isolation, which has a long culture cycle, sensitivity limited by sample collection, transportation, and handling, and is only targeted at a single disease. Serological tests may yield false negatives in neonates due to immature immune responses or transplacental maternal IgG interference. In addition, PCR, while highly specific, is typically targeted to known pathogens, and cannot detect unexpected or novel organisms. These methods also fall short in detecting co-infections or in cases where viral loads are low.^[16]^ However, metagenomic next-generation sequencing (mNGS) surpasses the limitations of traditional microbiology, which depends on culture and identification. This is because mNGS can directly sequence and analyze almost all microbial nucleic acids in a sample simultaneously, making it a cutting-edge tool for the rapid detection of pathogens in infectious diseases.^[17]^

In this case, the patient is a VLBWI born at 35 weeks and 2 days of gestation, despite broad-spectrum antibiotics and antifungal therapy, the infant experienced persistent fever with no clear bacterial or fungal etiology based on conventional tests. Repeated cultures were negative. These diagnostic challenges combined with the patient’s immunocompromised status and the need to detect possible uncommon or mixed infections prompted the clinical team to employ mNGS as a diagnostic approach. In addition, mNGS has the advantage of detecting co-infection, which is particularly useful in immunocompromised patients or neonates with complex clinical presentations, mNGS testing was chosen. The result diagnosed a CMV infection, which was subsequently confirmed by PCR. Given the potential for severe complications, early antiviral therapy was initiated. This case highlights the importance of advanced molecular diagnostic tools in neonatal complex infectious diseases and underscores the need for vigilant monitoring of CMV transmission in VLBWI receiving breast milk.

## 2. Case report

### 2.1. Case presentation

This case report presents a preterm infant diagnosed with CMV infection, managed in the neonatal intensive care unit. The patient, a preterm infant, was born at 35 weeks and 2 days of gestation as the 1st of twin gestation to a mother with severe preeclampsia and discordant fetal growth. The delivery occurred via cesarean section. The infant’s birth weight was 1040 g, and Apgar scores were 8 at 1 minute and 9 at 5 minutes. The amniotic fluid was clear, and the umbilical cord had a single artery.

### 2.2. Diagnosis and treatment

Immediately post-birth, the neonate was admitted to the neonatal intensive care unit due to prematurity, low birth weight, and respiratory distress, with initial diagnoses of neonatal respiratory distress syndrome, extremely low birth weight, prematurity, twin gestation, and small for gestational age. Initial interventions included umbilical vein catheterization, noninvasive ventilation support, surfactant replacement therapy, and feeding with preterm formula. Laboratory findings showed the serum CMV IgG level was significantly increased (191.732 AU/mL [ref: 0–10 AU/mL; ↑]), which was likely to reflect the transplacental metastasis of maternal antibodies, suggesting that the mother had a history of CMV exposure, but not active congenital infection. The initial breast milk CMV DNA load was <2000 copies/mL, which was in the low risk range. However, due to its underdeveloped immune system and poor virus clearance, it also poses a risk to VLBWI. Therefore, the lower CMV DNA load in the early postpartum period does not exclude the possibility of significant postpartum transmission, especially when the virus shedding increases with time. From the 4th day postnatal, the infant was fed with breast milk combined with a breast milk fortifier. The decision to resume breastfeeding was taken after normalization of infection markers and stabilization of the clinical condition. The breast milk CMV DNA level was monitored, and the benefits of breast milk in VLBWI, such as nutritional and immunological support, were weighed against the risk of recurrent viral transmission.

By the 15th day, a lumbar puncture was performed for treatment due to a ventricular enlargement rate >2 mm/week, and the cerebrospinal fluid (CSF) was tested. The CSF results were normal, and there was no bacterial growth.

**Figure 1.**
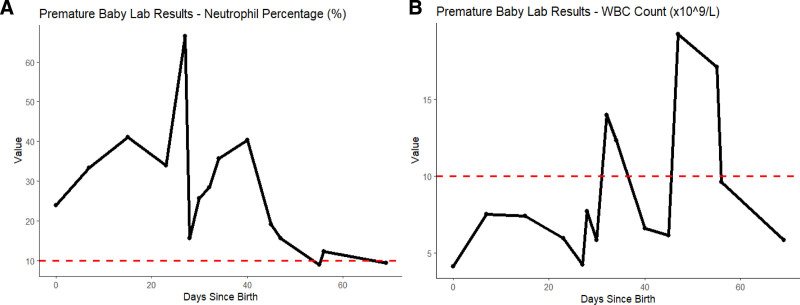
(A) Neutrophil percentage over time in patient. This graph illustrates the percentage of neutrophils in the blood of a premature baby from birth to 72 days of age. The *x*-axis represents the number of days since birth, and the *y*-axis shows the neutrophil percentage. (B) White blood cell (WBC) count over time in patient. This graph depicts the total white blood cell (WBC) count in the same premature baby across the same time period. The *x*-axis represents the days since birth, and the *y*-axis displays the WBC count in units of 10^9^ cells per liter. The dashed red line means reference threshold.

On postnatal day 27, the infant presented with hematochezia, the neutrophil percentage and white blood cell (WBC) count exhibited their first significant peaks on the 27th day (as shown in Fig. 1A and 1B). The spike in neutrophil percentage suggested a substantial inflammatory response, while the rise in WBC count further corroborated the activation of an infectious process. An abdominal ultrasound of the liver, gallbladder, pancreas, and spleen revealed portal venous gas. Additionally, an intestinal ultrasound showed thickened bowel walls, intramural gas, and pneumatosis intestinalis. These findings, combined with a bedside chest and abdominal X-ray, led to the diagnosis of NEC. The initial treatment plan included withholding oral intake, gastrointestinal decompression, and administration of cefoperazone-sulbactam (40 mg/kg twice a day intravenously) for antimicrobial therapy.

Thirteen hours after the onset of hematochezia, the infant developed a fever with a temperature of 38.5°C. Physical cooling measures were applied. The Infection marker test showed significant elevation compared to the initial levels at the onset of hematochezia. Then the antibiotic therapy was escalated to meropenem (20 mg/kg 3 times a day intravenously). After 9 days, the infection markers returned to normal, and the infant was fed breast milk again.

On the 45th day of life, the patient experienced a recurrence of elevated body temperature, accompanied by clinical signs such as a heart rate of 170 to 185 bpm and a capillary refill time of approximately 2 seconds. Peripheral blood examination (Table 1) indicated potential infection, leading to the administration of cefoperazone-sulbactam (40 mg/kg twice a day intravenously). Subsequent analysis of blood cultures and fungal cultures from oral secretions was conducted to rule out the presence of bacterial and fungal infections, with the results indicating no abnormalities detected.

**Table 1:** The results of peripheral blood examination.

Test	Result	Reference range
White blood cell count	6.16 × 10^9^/L	4.3–14.2 × 10^9^/L
Platelet count	188 × 10^9^/L	183–614 × 10^9^/L
Neutrophil percentage	19.2%	7%–56%
C-reactive protein (CRP)	14.28 mg/L	<10 mg/L
Serum amyloid A (SAA)	16.36 mg/L	<10 mg/L
Procalcitonin (PCT)	0.120 ng/mL	<0.05 ng/mL

**Figure 2.**
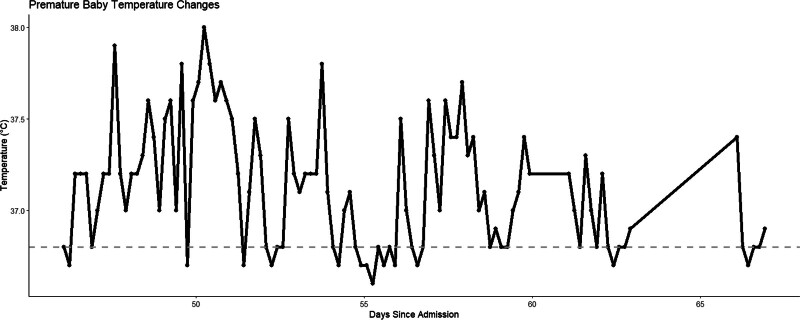
Temperature fluctuations in patient from 45 to 72 days. This graph shows the fluctuations in body temperature. The *x*-axis represents the number of days since admission, and the *y*-axis shows the temperature in degrees Celsius (°C). The dashed gray line at 36.8°C represents a reference value for the lower boundary of normal body temperature.

Starting from the 46th day, the infant exhibited persistent temperature fluctuations, with repeated episodes of rising and falling body temperature (Fig. 2). This sustained pattern of temperature instability was concerning and suggested an ongoing or unresolved infectious or inflammatory process. During this period, WBC count reached its second notable peak (Fig. 1B), suggesting a heightened immune response. This combination of elevated WBC count and recurrent temperature spikes pointed towards a persistent or worsening infectious process.

On the 49th day postnatal, the infant’s temperature remained elevated, a throat swab mNGS test was performed at this time. Long-term use of antibiotics can induce fungal infections, and fluconazole (3 mg/kg per dose intravenously) is given to prevent fungal infections. The patient exhibits enduring fluctuations in body temperature and has been administered cefoperazone-sulbactam for an extended duration as part of an anti-infective regimen, subsequently transitioning to piperacillin-tazobactam (75 mg/kg 3 times a day intravenously). CSF analysis showed the following results: glucose at 2.32 mmol/L, protein at 0.68 g/L, lactate dehydrogenase <25 U/L, and chloride at 120.50 mmol/L. Analysis of the CSF reveals a negative protein qualitative test, with the appearance was colorless and clear, effectively ruling out purulent meningitis. Chest CT and trachea computed tomography virtual endoscopy, demonstrate multiple inflammatory alterations in both lungs, thereby substantiating the diagnosis of pneumonia. Following antimicrobial therapy, the infection markers normalized, but the infant’s temperature remained persistently elevated.

To identify potential causes, assess immune deficiencies, and understand the body’s immune status, lymphocyte immunotyping experiments were conducted, a lymphocyte immunophenotyping analysis was conducted, showing the following results (Table 2). The increased T cell counts suggested enhanced cellular immunity, indicating possible viral or intracellular bacterial infections (Hepatitis B virus, CMV, Epstein–Barr virus, or tuberculosis). An immunoglobulin profile was performed, showing the following results (Table 3). Both IgA and IgG were below normal, indicating the possible presence of autoimmune diseases, congenital immunodeficiency diseases, viral infections, or infections by specific pathogens.

**Table 2:** Lymphocyte immunophenotyping analysis.

Cell type	Ratio	Absolute count cells/µL
NK cells	22%	1504
B cells	9%	579
CD3^+^ T cells/lymphocytes	66%	4398
CD3^+^CD4^+^ T cells/lymphocytes	26%	1773
CD3^+^CD8^+^ T cells/lymphocytes	34%	2306

NK cells = natural killer cells.

**Table 3:** Immunoglobulin and complement levels.

Test	Result
IgA	0.11 g/L
IgG	1.46 g/L
IgM	0.55 g/L
Complement C3	0.92 g/L
Complement C4	0.24 g/L

mNGS of a throat swab revealed the presence of *Mycoplasma pneumoniae* and CMV. Azithromycin (10 mg/kg once a day orally) was added to the treatment regimen for *M pneumoniae*, but the infant continued to have a fever after 3 days. Further testing showed a urinary CMV DNA at 6.24 × 10^4^ copies/mL (ref: 0–2000 copies/mL; ↑) and breast milk CMV DNA at 3.03 × 10^4^ copies/mL (ref: 0–2000 copies/mL; ↑), confirming CMV infection. Given that the child is a VLBWI with poor immune functions, recurrent fever, pneumonia, and confirmed CMV infections, ganciclovir (6 mg/kg twice a day intravenously) treatment was initiated. Thirteen days later, the infant’s fever resolved. At 72 days of life, the infant improved and was discharged, with continued oral valganciclovir (16 mg/kg twice a day orally) for CMV infection.

During the medication period, a full blood counts, WBC differential, platelet count, liver function, and creatinine clearance rate was performed weekly to assess whether there are adverse drug reactions. Monitoring blood CMV DNA viral loads weekly during treatment was conducted to evaluate efficacy. Fortunately, the child had a smooth medication process with no adverse effects and clear efficacy. The medication was used for a total of 6 weeks before discontinuation.

### 2.3. Outcome and follow-up

During the course of medication and subsequent to the cessation of pharmacotherapy, assessments including fundoscopic evaluation, brainstem auditory evoked potential testing, and cranial ultrasonography were conducted to gauge the extent of injury and its progression. Neurodevelopmental evaluations and auditory assessments were performed at 6 months and 1 year, both of which presently exhibit normal results. The presence of any long-term sequelae necessitates ongoing follow-up.

The younger sister was born weighing 2370 g, hospitalized for 8 days, and improved before discharge. Since then, exclusive breastfeeding has been upheld. The urine CMV levels were recorded to be below 2000 copies/mL (ref: 0–2000 copies/mL) at both the 2 and 3-month postnatal intervals, indicating the absence of CMV infection.

## 3. Discussion

**Figure 3.**
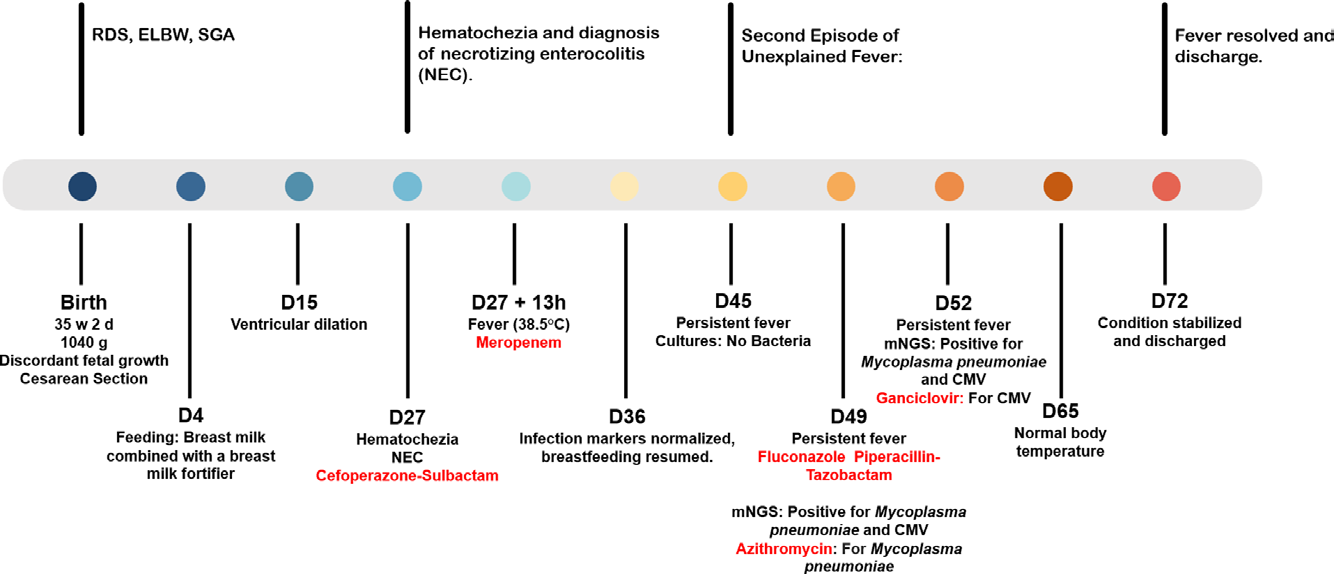
Timeline of a preterm infant diagnosed with *cytomegalovirus* (CMV) infection. CMV = Cytomegalovirus, ELBW = extremely low birth weight, NEC = necrotizing enterocolitis, RDS = respiratory distress syndrome, SGA = small for gestational age.

This case describes a VLBWI who developed persistent fever, NEC, and pneumonia during hospitalization. Traditional diagnostic methods including blood cultures, fungal tests, and CSF analysis failed to identify a pathogen. mNGS of throat swab ultimately revealed CMV and *M pneumoniae* infection. High CMV DNA levels in urine and breast milk confirmed pCMV transmission. Early antiviral treatment with ganciclovir led to clinical improvement and favorable short-term outcomes. Figure 3 illustrates the 72-day course of treatment, highlighting key events and therapeutic interventions.

Breast milk contains numerous immunologically active components – such as immunoglobulins, oligosaccharides, growth factors, and lactoferrin – that support gut microbiota development and enhance neonatal immunity, particularly in VLBWI.^[18,19]^ However, it can also serve as a major route of pCMV transmission in preterm infants.^[20]^ CMV seropositivity among women of childbearing age ranges from 50% to 85%, and in up to 96% of seropositive mothers, the virus reactivates during lactation and is shed into breast milk.^[21]^ CMV DNA levels in breast milk are typically low or undetectable in colostrum but rise and peak between 4 and 8 weeks postpartum before gradually declining.^[22]^

In this case, the infant was breastfed and had elevated CMV IgG with negative IgM in the first 3 weeks of life, suggesting passive maternal antibody transfer and excluding congenital infection. CMV DNA was initially low in breast milk but increased significantly by week 7, indicating viral reactivation. The infant’s urine tested CMV DNA-positive at 4 weeks, aligning with the typical 4 to 7 week incubation period for postnatal infection in preterm infants.^[21]^ These findings strongly support the likelihood of breast milk–acquired pCMV infection in this case.

The clinical manifestations of pCMV infection span from asymptomatic cases to multi-organ dysfunction affecting the liver, lungs, blood, and nervous system. VLBWI may also develop complications such as sepsis, bronchopulmonary dysplasia, and NEC.^[7,8,10,13,23]^ The smaller the gestational age, the higher the proportion of symptomatic infections, likely due to an underdeveloped immune system.^[24]^ Moreover, CMV infection has been reported to protect against unrelated pathogen infections and enhance responses to vaccination and antigen stimulation.^[25,26]^ CMV infection may enhance the immune response to combat infection by increasing pro-inflammatory cytokines in circulation and activating T cells and natural killer cells.^[27]^ Intriguingly, human CMV-vectored vaccines emerge as a promising innovative vaccine platform capable of harnessing these advantages by stimulating robust T and natural killer cell responses elicited by the vaccine.^[28,29]^ But studies on the impact of CMV infection in childhood and on pediatric vaccine responses remain limited.^[27]^

In this case, the diagnostic process was complicated by persistent fever, fluctuating inflammatory markers, and nonspecific signs such as NEC and pneumonia, but no neurological symptoms. Conventional investigations, including blood and fungal cultures, CSF analysis, and imaging studies, failed to identify a causative pathogen. Despite temporary normalization of infection markers, the fever persisted, raising concern for unresolved infection or atypical etiology. Given the lack of microbiological confirmation and limited response to empirical antibiotics and antifungal agents, differential diagnoses included atypical bacteria, congenital or postnatal viral infections, and immune dysregulation.

At this point, mNGS was employed due to its ability to detect a broad range of pathogens in an unbiased manner. mNGS successfully identified CMV and *M pneumoniae* in a throat swab sample, both of which were subsequently confirmed by PCR and consistent with the clinical presentation. Compared to traditional CMV diagnostic methods – such as targeted PCR of urine, saliva, or breast milk – which may fail during latent phases or when viral load is low,^[30]^ mNGS enables simultaneous detection of multiple pathogens in a single test, offering distinct advantages in complex neonatal cases.^[31]^ Expert consensus also recommends mNGS as a second-line tool when conventional methods fail in patients with unexplained, infection-related symptoms.^[32,33]^

Following confirmation, antiviral therapy with intravenous ganciclovir was initiated promptly, resulting in the resolution of fever and clinical stabilization. Early treatment of CMV in neonates, particularly VLBWI, is essential to reduce the risk of long-term sequelae such as hearing loss, developmental delay, and neurological impairment.^[34]^

CMV infection has been implicated as a potential contributor to the pathogenesis of NEC, particularly in VLBWI. The studies have reported an increased incidence of NEC in neonates with CMV infection, suggesting that viral-mediated intestinal inflammation and endothelial injury may predispose the immature gut to NEC.^[13]^ In the present case, the diagnosis of NEC was based on typical clinical and imaging findings, including hematochezia, bowel wall thickening, and pneumatosis intestinalis. Although a direct causative link cannot be definitively established, the co-occurrence of CMV infection and NEC in this infant aligns with emerging literature and raises the possibility of a contributory role. Further research is needed to clarify the mechanistic relationship and determine whether early identification and treatment of CMV can mitigate NEC risk in vulnerable neonates.

In addition, long-term follow-up of CMV infection in VLBWI is crucial. Some infants with CMV infection may develop sensorineural hearing loss within the first 2 years of life.^[35]^ Therefore, multiple hearing reevaluations should be conducted within the first 2 years after birth (42 days, 3 months, 6 months, 1 year, 1.5 years, and 2 years). VLBWI with acquired CMV infection may have neurological complications or eye-related diseases, so they should also receive annual neurodevelopmental assessments and ophthalmic examinations.^[36]^

In summary, VLBWI with immunodeficiency who acquire CMV infection through breastfeeding often present with latent infections and lack specific clinical symptoms, leading to misdiagnoses. When traditional detection methods fail to provide effective evidence for infectious diseases, mNGS should be promptly utilized to aid in etiological diagnosis, thereby enhancing the accuracy and timeliness of infectious disease diagnosis and reducing the risk of sequelae. The long-term prognosis for the patient in this case remains to be observed, necessitating continued follow-up.

## Author contributions

**Conceptualization:** Jihong Liu.

**Data curation:** Huimin Li, Menghan Jia.

**Investigation:** Li Guo.

**Writing – original draft:** Chunhui Zhao, Huimin Li, Jihong Liu.

**Writing – review & editing:** Li Guo, Menghan Jia, Jihong Liu.
